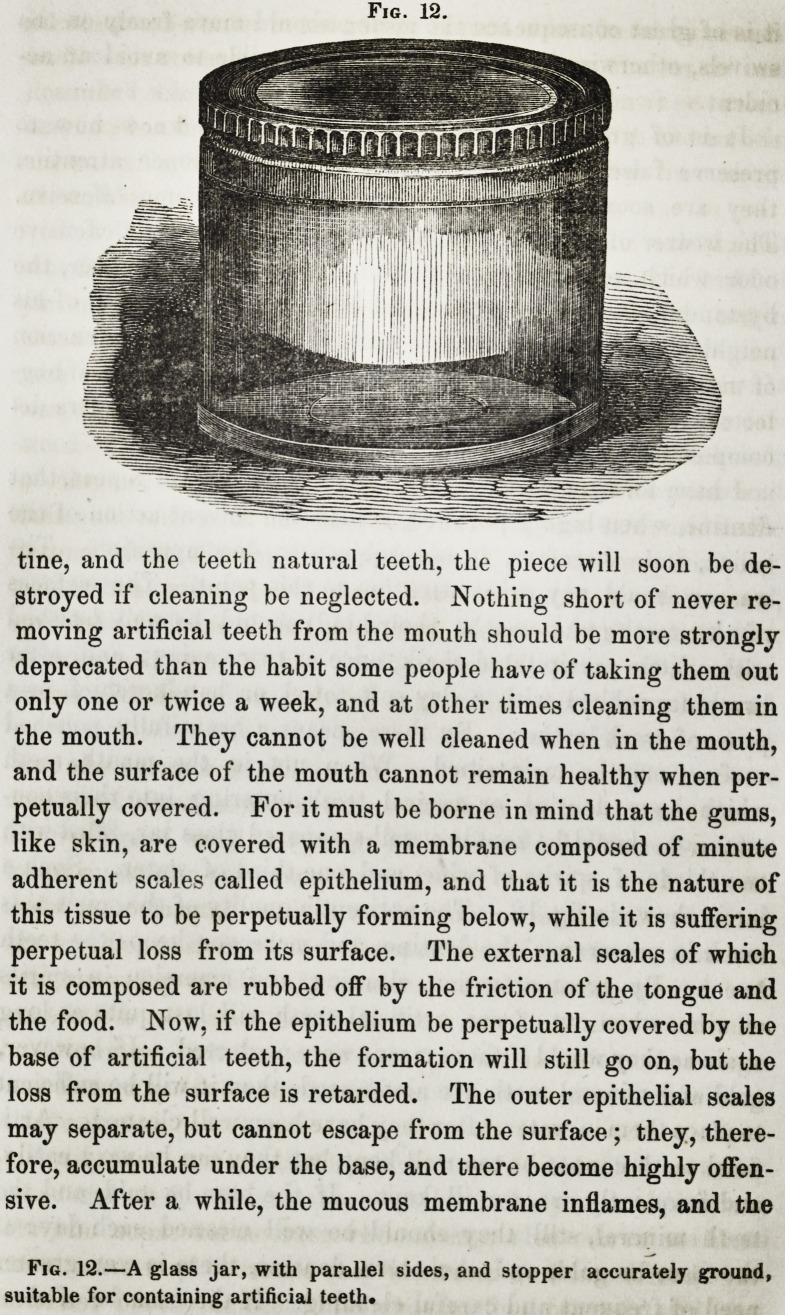# Instructions in the Use and Management of Artificial Teeth

**Published:** 1854-07

**Authors:** John Tomes

**Affiliations:** Surgeon Dentist to Middlesex Hospital, London.


### SELECTED ARTICLES.

Some years ago we published in the Journal the lectures of Mr. Tomes on Dental Physiology and Surgery, corrected from the London Medical Gazette. The last lecture of his course was on artificial teeth. This we did not receive at the time of the publication of the others. It has recently appeared in a small 12mo volume, and we now take the liberty of giving it to our readers.—*Eds.*

### ARTICLE XIV.

*Instructions in the Use and Management of Artificial Teeth.* By John Tomes, F. R. S., Surgeon Dentist to Middlesex Hospital, London.

I promised, before concluding this course of lectures, to give you some account of artificial teeth—their use, their construction and their management. I will now redeem my pledge; but I must be brief, for we have but one lecture at our disposal, and the subject is a large one.

Most of you are about to enter on the path of general practice, and in the exercise of your calling, will have, from time to time, to prescribe the use of artificial teeth to those of your patients who have lost the natural organs of mastication, and are suffering from dyspepsia, consequent on the food being carried into the stomach before it has undergone a sufficient amount of comminution. It is fit, therefore, that you should possess some general information on the subject, and that you should be able to instruct your patients in the management of the apparatus which you have found it necessary to recommend for their use. I shall therefore endeavor to give you such information as the wearer of artificial teeth will find it advantageous to possess.

The duration of human life is proportioned to the perfectness or imperfectness with which the various functions that collectively constitute life are performed. These functions are the work of organs on materials submitted to their action. It is obviously necessary that before organs can work well, they shall be well formed, and fully endowed with power to work. But it is equally necessary that the materials submitted to them shall in all respects be such as they can readily, and without unusual effort, act upon.

Digestion is one of these functions, and it is one of the first and most important; for without it others can go on but for a while, and if it be deranged, others become more or less disordered as a consequence.

Digestion consists in the reduction of the various articles taken as food to a pultaceous mass called chyme, from which the more purified nutriment, chyle, is eliminated. This is carried into the circulation, through a set of vessels destined for the purpose, mixes with and becomes blood.

The reduction of food to chyme is in great part a chemical action: a fluid is furnished by the stomach which dissolves the food.

Substances taken as food, irrespective of their relative solubility, are dissolved by the gastric fluid, quickly or slowly, in proportion to their degree of permeability or comminution; or, in other words, in proportion to the surface exposed to the action of the solvent. So it happens that a solid morsel swallowed whole may remain in a healthy stomach many hours before it is dissolved, while had the same morsel been crushed, or broken up into many pieces, and in that process mixed with saliva, and then swallowed, it would have been reduced to pulp in an hour.

In the one instance, the function of digestion is duly performed; in the other, it is retarded.

Hence it is of paramount importanee that the food, before it is introduced into the stomach, should be retained in the mouth, while it is properly crushed, divided, and thus rendered pervious to the gastric juice, in other words, that mastication should be perfect. To effect this purpose, we are provided by nature with a special apparatus, with suitable crushers—with teeth.* To the dentist is entrusted the care of these important organs, to keep them in repair, and to replace them when lost.

I have told you how to preserve them, how to repair them:† I will now tell you how they may be replaced. How, on the one hand, you may preserve the probabilities of life by preserving the organs of mastication; how, on the other, you may regain the lost probabilities by forming efficient substitutes for the lost organs.

But there are other, though less important, yet sufficient reasons, why we should use our best endeavors to preserve our natural teeth; and, when lost, to replace them by artificial teeth.

Teeth—and especially front ones, natural or artificial—are necessary to distinct articulation; and we owe it to ourselves, and also to those with whom we converse, that we should, if

*The admixture of saliva with the food is necessary, otherwise mincing would answer the purpose of mastication, which is not found to be the case.

†Lectures on Dental Physiology and Surgery.

possible, be readily and distinctly understood—that our utterance should be perfect.

The absence of teeth deprives the face of much of its character, and the appearance of old age is imprinted at a period when, under ordinary circumstances, health and strength remain. In this case it is due to ourselves, and more especially to those about us; and also, though in a less degree, to all with whom we meet, to preserve our natural and healthful appearance by all available means. It is not natural for young or middle-aged people to be without teeth, and it is not unnatural for old people to have them.

We are in no danger of over-rating the value of the dental apparatus, so long as we consider it as one only of the many parts that compose the human system, the well-being of each of which is necessary to the well-being of the whole, and therefore to health and comfort, and through these to longevity.

I will now endeavor to give you some account of the various kinds of artificial teeth, of the principles on which they are severally constructed, and of their applicability. I cannot, however, give you a detailed description of their manufacture; neither would the description be useful if I did. The constructive process must be seen to be understood, and practiced to be learned. Nor can I enter into details on the modifications in form that the peculiarities of individual mouths may require. To do so would occupy many lectures instead of one. On the contrary, I can only sketch briefly the general principles of construction, and in doing so, shall confine my remarks to complete, or nearly complete, sets, or half-sets, unless it be otherwise stated; and my remarks will have especial reference to the mode of use, and methods of preservation.

The natural teeth have fangs, which pass through the gums, and are socketed in the jaws. In artificial teeth, we must have a part corresponding to the fangs, but here it must be spread over and rest upon the gums, and through it, as through the fangs of natural teeth, the pressure of mastication must be communicated to, and borne by, the jaws. This part we shall call the *base*, or *foundation*, since from it the crowns of the false teeth must rise. (Fig. 1, *a*.) The base is an essential part of all artificial teeth, whether they be few or many; and upon the accuracy with which this fits the gums, will the usefulness of the imposed teeth depend. Indeed, unless it fits tolerably, the teeth cannot be worn; and, for this obvious reason, that the pressure of mastication will be communicated to those parts only of the gums on which the base bears. If the area of these be small, the parts will be bruised; if they be still smaller, they will be cut. The greater the area over which the pressure is diffused, the less will it be felt; the smaller, the more. We all know what would be the consequence if the area were reduced to an edge or a point; yet there are not wanting instances where, from inattention to these simple facts, the bases of false teeth are so badly constructed, that the gums are bruised or cut the first time they are worn, and this from the ill-fitting or insufficient size of the base. Hence, in estimating what would be the probable value of artificial teeth in any particular case, the first consideration will be, whether the base can be made to fit perfectly, and whether of sufficient superficial extent. If both of these points can be, and are attained, the base will, when pressed on the gums, bear pretty equally over the whole surface it covers, and when so pressed, will squeeze from between itself and the surface of the gums, both the saliva and the air; and will then be retained in its position with considerable force by the at-

Fig. 1.

Fig. 1.—Artificial teeth for an edentulous upper jaw, showing, *a* the base of gold; *b* the side block which takes the place of molar teeth; *c* the front teeth; *d* the right side of the base, with pins soldered to the base for fixing the teeth and the side block.

mospheric pressure acting on the non-fitting surface only. And, further, it may be foretold, that if the subsequent stages of construction are successfully conducted, the new will be very useful substitutes for the lost teeth.

The base of artificial teeth is usually formed either of sheet-gold, or of dentine, or ivory, as it is more commonly called,* the dentine of the hippopotamus, or of the walrus-tooth, and by the following means:—Beeswax, previously softened by immersion in hot water, or exposure before the fire, and well kneaded, and then placed in a horse-shoe shaped tray of suitable size, is introduced into the mouth and carefully pressed against the gums until they are perfectly imbedded. The tray of wax is then as carefully withdrawn, and, if successfully, it will present a perfect mould, or counter cast of the gums. Into this plaster of paris is poured, and allowed to set; after which it is removed from the wax by softening the latter. The plaster then presents a cast, a fac-simile, both in size and form of the gums, supposing, of course, the mould to have been correct.

It was usual in my practice, and I believe in that of other dentists, to assume the cast of the gums obtained by the pres-

* The material employed for making the base is obtained either from the tusks of the walrus, hippopotamus, sometimes the teeth of the sperm whale, and now and then portions of the tusk of the elephant are used. These teeth or tusks are composed of two or three substances; the central substance is dentine, and forms nine-tenths of the whole tooth; external to this, in certain parts of the tooth, is the enamel; and external to the enamel is a third substance, called cementum, which is the softest of the three. In the tusks of the walrus, whale, and elephant, the enamel is absent in teeth fitted for dental purposes, and the cementum coats externally the dentine. Teeth so constituted are in commerce called ivory, which term includes both the dentine and cementum. But as the latter substance is not suitable, in consequence of its comparative softness and disposition to discolor, and is, therefore, rejected, the term ivory would not definitely express the nature of the material employed in making artificial teeth. I have, therefore, adopted the term dentine, as being expressive of the material used, and as being that by which this substance is designated in scientific writings. Dentists, when speaking of this substance, usually call it bone, and sets of teeth made of dentine they call bone-sets, although the material differs very consiberably from bone, in possessing the qualities of great hardness and compactness, the absence of which renders the latter substance totally unfit for dental purposes.

ent process to be correct, and upon that faith to proceed to construct the teeth to fit the plaster cast, until about four years since, when I had the good fortune to discover means whereby the correctness of the cast could be readily tested. Since this time, I have always availed myself of the test previous to constructing the teeth.

The means I allude to, with other appliances for teeth-making, formed the subject of a patent in 1846. It consists in the compounding of a material like in composition to extremely hard sealing-wax, but which is soft and plastic at the temperature of boiling water, though hard and unyielding at that of the human body. This material, when softened, is moulded on the plaster cast into the shape of the required teeth. Thus we have, at a very trifling cost of time, a model of the new teeth, on which, by the aid of a little hot water, we can work any required changes, should it, on being placed in the mouth, need any. And this, of course, will depend on the faithfulness of the cast on which it has been moulded. If the cast be correct, the model will fit equally well both the cast and the mouth; but should the cast be faulty, the model made on it will not fit the mouth, whereby we discover the error in the cast, and proceed to its correction. The faulty cast is thrown away, and the composition model is slightly softened by immersion in hot water. When in this state, it is carefully moulded to the surface of the gums, and then allowed to harden. When hard, it is again put in the mouth, and if found to fit, is used to furnish a plaster cast in the same manner as the bees-wax mould did in the first instance. By these means we obtain a known *perfect* cast, to which we may make the new teeth without fear of failure. Should gold be chosen for the base, casts in metal, zinc, or brass, are made from the plaster cast, and from these again counter-casts, or reverses in lead are made, between which and the cast, gold plate is hammered, until it has assumed the form, and fits perfectly to the surface of the cast; and, of course, also to the gums.

If dentine be chosen for the base, it is usual to cover the plaster cast with red pigment, and to place upon it a block of dentine in the position it is required to take when fitted. The block will at first touch only at, or on two points, and these will be marked by the adhesion of a little of the pigment. The points so indicated are cut away with small tools, similar to those used by engravers. The contact is renewed, and the reddened points again removed. In renewing the contact between the block of dentine and the paint-covered cast, great care should be taken to keep the two in the same relative position as on each preceding occasion. This tedious and somewhat uncertain process is repeated again and again, to the extent of many hundreds of times, till the block is at last cut to fit the surface of the cast. The superfluous portions are then removed, and the base so made is prepared for the reception of the teeth.

In my own practice, the base is carved by a patent machine, which altogether supersedes the hand carving and the use of pigment. A model of the required teeth is made in the moulding composition, and this is fixed in the machine, and then copied into dentine with much saving of time, and without liability to error.

For the invention of this instrument, I had the honor to receive a gold medal from the Society of Arts.

There are, however, a few workmen to be met with, who, from great practice and a considerable amount of ingenuity, produce results that can scarcely be surpassed. But such men are not numerous; hence, their services cannot at all times be commanded.

The base, so far as its gum-fitting surface is concerned, having been finished, we have next to select the teeth which the base is destined to carry.

Teeth used in making artificial teeth are of three kinds: human teeth, mineral, and carved teeth—that is, teeth carved out of dentine. The latter, when dentine is used for the base, are carved out of the same block in one piece, as shown in fig. 2. When natural or mineral teeth are selected, they are fixed to the base by pins.

In speaking of artificial teeth, dentists divide them into front teeth and side blocks. The front teeth are like, and have the same names as the natural teeth, including the bicuspides (fig. 1, *c*;) while those corresponding to the molar teeth are made in one continuous piece, and are called the side-blocks of the piece (fig. 1, *b*.) I should here tell you that the teeth, whether few or many, with their base, when spoken of as a whole, are termed by dentists a *piece*—an upper or under piece, as they may be for the upper or lower jaw.

Fig. 2.

The teeth are fixed to the base by pins passing through, or nearly through the centre of each tooth, and soldered to the gold, or riveted through the dentine, according as the base may be composed of the one material or the other (fig. 1, *d*.)

Should a tooth, when in wear, come off its pin, it may be temporarily refixed by wrapping a little fine silk round the pin, and then replacing the tooth.

Fig. 3.

The American and French dentists frequently use teeth, from the back of which small platinum pins or bands project, and these are soldered to small vertical plates of gold, which have been previously fitted to the base, as shown in fig. 3. In this country, mineral teeth made on the above plan, are used in

**Fig. 2.—Artificial teeth for an edentulous upper jaw, with the base and the teeth carved out of a solid block of dentine, and retained by atmospheric pressure without springs or clasps.**

**Fig. 3.—Three teeth for the upper jaw: *a*, the base; *b*, a vertical plate of gold soldered to the base, and perforated to receive the pins of an American tooth; *c*, the tooth, with the pins projecting from the inner surface; *d* and *e*, two teeth soldered to the vertical plates; *f*, a band for clasping a molar tooth.**

in cases where, from the relations of the upper and lower teeth, peculiar strength is required. Should a tooth break off the gold, the back usually remains standing, as shown in fig. 4, *b*; against which a little white wax may be moulded, if a dentist be not at hand to make the necessary repair. I once met with a lady who had, for six months, worn wax teeth moulded on to the gold backs of a set of American teeth. She informed me that the wax required to be renewed every third day, and that her friends had not detected the injury the teeth had sustained.

Fig. 4.

The piece having been so far finished, the *bite*, or closure of the upper and under teeth, must be adjusted; that is, the teeth of the two jaws must be so adjusted that, on closing the mouth, all meet at the same moment. Should the teeth of one side meet before those of the other side of the mouth, the piece will be displaced at each attempt at mastication; or if the more posterior parts meet before the anterior teeth, the same result would occur. If, when the teeth are put in, the error is considerable, the more prominent parts will be readily seen, and may be removed; but when the bite or closure is nearly perfect, recourse may be had to pigments. The upper or under teeth, as the case may require, must be covered by the paint, and the points of contact, when marked by closing the mouth, removed by the graver or file, until, on trial, all parts receive equally the color from the opposing teeth.

In my own practice, I use the composition I have before spoken of. The piece is moulded in this, and the parts corresponding to the side-blocks and teeth slightly softened by heat, so that when the mouth is firmly closed, those parts which are too prominent will yield till the proper level is obtained. The bite thus gained is copied in the artificial teeth.

**Fig. 4.—A view of the same teeth as shown in fig. 3, but placed in the mouth: *b*, the vertical plate of gold for the attachment of a tooth; *d* and *e*, teeth fixed to similar plates; *f*, the band encircling the molar tooth.**

Artificial teeth are retained in the mouth by three different plans: (1st) by spiral springs attached by their ends to the pieces of the two jaws, when the set is complete, as shown in fig. 5, or when the under teeth are perfect, to caps fitted to these teeth.

FIG. 5.

The springs themselves are made of gold wire, twisted spirally round a small piece of cylindrical steel. They are fixed to the teeth by a swivel or loop, through which a pin passes into the base or to the blocks; while the swivel itself terminates in a piece of wire, which exactly fits into the interior of the spring, into which it is pressed. These parts are delineated in fig. 6. With this arrangement the springs are readily detached, even by the patient. Should the spring fit too loosely on the swivel, a little floss silk should be wrapt round the latter before pressing it into its place in the spring. And this, too, may be done by the patient, should a spring accidentally leave the swivel.

The pin on which the loop moves is either passed through the side block, as in fig. 6, *a*, or screwed into or soldered to the gold base, as shown in fig. 5, *d*.

(2nd.) By clasps, or bands, of elastic gold, passing partly round natural teeth. The clasp is attached in a part only of

**FIG. 5.—A complete set of artificial teeth shown in the position they occupy in the mouth; *a* and *b*, the side blocks of the upper and lower teeth; *c*, the spring in its proper position when the mouth is closed; *d* and *e*, the pins by which the spring is attached to the upper and lower teeth; *f* and *g*, the front teeth.**

its length to the base, the remaining portion is left free, and

Fig. 6.

springs open to receive the tooth. If at any time the clasp does not firmly embrace the tooth, it is only necessary to bend the free portions towards each other to make it do so; it will then again take firm hold, and present the appearance shown in fig. 7.

Fig. 7.

(3rd.) By the pressure of the atmosphere. The fitting surface is so accurately fitted to the surface of gum, that the saliva and the air are excluded, whereby the pressure of the atmosphere acting only

Fig. 6.—Spiral springs, with the apparatus for attaching them to the teeth: *a*, the pin which passes through the loop or swivel *b*, into the block *d*; *c*, the spring. In the left hand figure the several parts are shown detached, with dotted lines indicating their position when fixed. In the right hand figure, they are shown in their proper positions.

Fig. 7.—Three artificial teeth for the upper jaw, on a gold base, fixed in the mouth by a band: *a*, the gums; *b*, the three new teeth; *c*, the band, which, from its situation in the back part of the mouth, is not seen.

on that surface of the teeth exposed to the tongue, holds them in tight contact with the gums. Such a piece is shown in fig. 2.

Teeth on this principle, though the most difficult to construct, are, in some respects, the best kind when well constructed, seeing that they are, in great part, independent of any remaining natural teeth of the same jaw, and also of those of the opposite jaw.

It may be stated, generally, that when a complete set of artificial teeth, both for the upper and lower jaws is required, spiral springs are best adapted for their retention in the mouth; but if two or three teeth only are worn, clasps will prove most effective. Then, again, when a number are required for one or both jaws, while a few natural teeth remain; or even supposing the teeth are all gone, and the gums are favorably shaped, the artificial teeth may be retained by atmospheric pressure, or *suction*, as it is sometimes called.

In advanced age, the alveolar ridge, which supports and gives convexity to the gums, is in many individuals completely removed, and the roof of the mouth rendered quite flat. In such cases, teeth on the pneumatic principle will not be steady, but, on the contrary, they will glide about just as you may have seen two flat metallic surfaces, when inclined to a slight angle, slide readily off each other even by their own weight, though they required considerable force to separate them when applied at a right angle to the surfaces in contact. From these facts you will readily infer, that teeth so made will, if fitted with perfect accuracy, be effective in proportion to the amount of surface presented in the base and to the convexity of the gums.

The amount of atmospheric pressure will, of course, be proportioned to the surface of the base, and the freedom from lateral sliding in proportion to the convexity of the gums, unless there be teeth remaining in the jaw to steady them.

Pneumatic teeth are usually made of dentine, while those retained by clasps commonly have a gold base. Sometimes the base is made of dentine, and fitted round or between remaining natural teeth, and is thus retained. Then, again, teeth may be constructed to be retained by a combination of two of these plans.

Indeed, the combinations and modifications of plans available in the construction of artificial teeth are very numerous, and upon the successful adaptation of these to special cases does the usefulness of the dentist depend. No two mouths are exactly alike, and hence no two admit of precisely the same form of teeth; out of this endless variety in foım arises the difficulty of producing an equally successful result in each individual case.

The base having been completed, the teeth mounted and fixed, and the bite adjusted, the teeth must be given to the patient for wear, who must be directed to return on the following day should the mouth feel sore. If, when your patient returns, you find, on inspecting the mouth, that the base of the teeth has pressed on one part more than on another, and caused redness, the base at that part must be reduced by filing or it must be bent away from the injured part; and these operations of adjustment must be repeated from time to time till the teeth become easy; always taking as your guide the state of the mouth rather than the statement of the patient.

You might, at first thought, suppose that artificial teeth, when well made, would require no after adjustment to the mouth; and in many cases they do not—in others they require but very little: yet, again, they may require a great deal; and for the following reasons:—The base may press equally on all parts, but all parts may not bear pressure equally well. Then again, some parts of the jaw may be covered with a greater thickness of gum than others. Under pressure the thicker parts of the gum will yield, and leave the thinner to sustain the pressure that should be equally distributed over the whole. The points so pressed on will necessarily become sore, unless the piece be adjusted to relieve them.

The first effect, on putting in a complete set of artificial teeth, is most unquestionably great discomfort; the mouth feels filled, the speech rendered difficult and indistinct, and mastication impossible: yet, within a fortnight, or three weeks at most, and often within even a week, all those difficulties vanish, and the patient tells you he could not do without new teeth. Distressing nausea is amongst the occasional early consequences of wearing artificial teeth, but this also subsides with a little patience.

To masticate well with false teeth requires both time and perseverance, the ability being acquired sooner or later in proportion to the aptitude of the individual. But all may acquire it if the teeth be well made, and properly adjusted so that pressure on them does not produce pain.

The patient should, however, return to the dentist whenever the teeth give pain from pressing more on one part of the gum than on the other, that the unequal pressure may be removed before the surface of the gum has become abraded. To persevere in wearing teeth which press unequally on different parts of the mouth is in every way disadvantageous, and prolongs unnecessarily the period of discomfort.

There are a few persons, however, whose jaws are so formed that sufficient available bearing surface for the base can scarcely be found. There are others, again, in whom the lining membrane of the mouth is so irritable, either naturally or from habits of intemperance, that the presence of artificial teeth cannot be borne—or, at least, without great effort. But if the effort be made and continued, and the teeth are good in construction, and well adjusted, success, even in the most difficult cases, will certainly ensue.

In all cases, however, whether the teeth be few or many, whether for one jaw or both jaws, the construction and adjustment must be left to the dentist, who will at all times avail himself of any suggestions the patient may offer, if they be such as can be adopted with advantage.

It is particulary desirable that the patient should place himself wholly in the hands of the dentist, who, if unshackled with conditions, will be in a position to do the best he can to make him comfortable. The difficulties of making and adjusting artificial teeth really well, are great; and the first impression a new set of teeth produces when put into the mouth is so strange, that great confidence is required on the part of the patient, otherwise he loses hope, embarrasses the practitioner with exaggerated complaints, and thereby endangers the success of the operation.

Artificial teeth must be regarded by the wearer as tools, the use of which have to be learned by patient trials. The first time you take up a joiner's plane, you cannot work it, nor would you expect to do so without previous practice; so, with artificial teeth, you have no right to expect to masticate effectively with them until by practice you have learned their use.

I would recommend that patients before they wear new teeth should carefully examine them in their several parts, and actions, and thus learn how they should be used, and what is to be expected of the teeth and what of themselves in acquiring the art of masticating with artificial teeth. If this expedient be adopted, many ill-conceived attempts, and consequent failures productive of disappointment will be avoided.

Whatever may be the construction of the teeth, some little care is required in putting them into, and removing them from the mouth. It is by no means uncommon for those who are unpracticed in their use to do them serious damage in the one or the other of these operations. In sets of teeth retained by springs, the teeth themselves seldom receive any material injury, but the springs are frequently damaged, and not uncommonly rendered worse than useless by treatment which the wearer does not know there is any occasion to avoid. The following directions are given with the hope of lessening the frequency of such accidents, and they will be accompanied with a description of the manner in which the injuries are usually produced; so that not only may the proper methods of procedure be seen, but also the faulty ones.

Teeth which are retained by clasps similar to those which are represented in fig. 7, should be held with the thumb and finger resting on the sides of the teeth, and placed in a line with their intended position in the mouth, in the manner shown in fig. 8.

Fig. 8.

Fig. 8.—The three teeth as in figure 7, with the manner of holding them in placing them in the mouth: *a* the gums; *b* the base of the teeth; *e* the band; *c* the forefinger; and *d* the thumb.

The clasps will then pass around the extremities of the crowns of those natural teeth they are destined to embrace. When, by the aid of a mirror, they are seen to occupy that position, the teeth should be gently pressed till the base of the apparatus comes in contact with the gum. The base itself should then be pressed with the thumb, or finger, firmly on the gum.

If the artificial teeth occupy a space on each side of the mouth, the two hands should be used in putting them in, and the two thumbs in pressing the base on the gum.

These directions apply more particularly to the teeth for the upper jaw. In those made for the lower jaw, the thumb and finger should be used, but with the finger placed against that side of the teeth which lies next to the tongue. When the teeth occupy one side of the mouth only, it is best to use the hand of the corresponding side in placing them.

It must, however, be always borne in mind, that no force should be used, otherwise the teeth will be injured; for, if on making an attempt to put the teeth in the mouth they will not with gentle pressure readily pass into their place, the position is incorrect—they should be withdrawn, and sufficient time taken to get them in the proper position before again attempting to press them into the mouth.

The difficulties which are implied to exist by the directions which I am giving are confined to the first few days of wear. Those who have become accustomed to artificial teeth of this kind can put them in not only without the aid of a mirror, but also without the presence of light. They may, after a little practice, be taken out and returned to their place without the bystander becoming aware that his neighbor has other than natural teeth.

In removing teeth constructed on the foregoing plan, the finger nail or nails should be placed between the edge of the clasps and the gums, and then, by moderate pressure, the teeth may be withdrawn without any fear of injuring them. If the teeth extend to each side of the mouth, care should be taken to move the two sides of the teeth at the same time, otherwise the base may be bent out of shape.

Teeth retained by spiral spring require considerable care in putting them into the mouth. The wearer not unfrequently injures or entirely destroys two or three pairs of springs by bad management, before experience has taught the manner of avoiding such accidents. The proper position of the springs when the teeth are in, and the mouth is closed, is shown in fig. 5, and any deviation from that position will be attended with injury to the apparatus. If, for instance, a spring should get into the position shown at fig. 9, it will be so damaged, if not absolutely broken, that its action will ever after be imperfect; or if it should be allowed to project forwards towards the lips, great inconvenience will be felt, and the spring, if not speedily released, will most likely be permanently injured.

Fig. 9.

In order to avoid these unpleasant accidents, one or other of the following methods of putting the teeth into the mouth may be adopted:

In one method the upper and lower teeth should be placed with the masticating surfaces in contact, and with the springs in the position shown in fig. 5; the forefingers should then be placed over the upper and the thumbs under the lower teeth. In this manner the upper and lower teeth can be held firmly together: when so held, one side should be passed a short distance within the lips, and with it the cheek pressed outwards. By this means the mouth will be stretched sufficiently open to allow the other side of the teeth to be introduced without any fear of the spring becoming entangled with the lips, which, but for this precaution, would probably pass in between the spring and the teeth.

Having once got the teeth fairly into the mouth, they will almost of themselves find their proper position on the gums. However, it is desirable to press the base well into its place before attempting to close the mouth.

**Fig. 9.—Side view of a set of teeth, with *c* the spring bent in a double curve, and injured at *d*; *a* and *b* the upper and lower side-blocks.**

The second method to which I alluded is effected in the following manner. Instead of placing the upper and lower teeth in the mouth together, the two parts of the set may be put in one after the other.

The upper part of the set may be first pressed lightly into its place and held there by the help of the tongue. The lower division will then project from the mouth, and the springs connecting the two will remain straight, or nearly so. The second step of the process—that of placing the lower teeth—needs some little care, or the springs will suffer more or less injury in the operation. The forefingers should be placed on the masticating surface of the teeth, in doing which the springs will be

Fig. 10.

**Fig. 10.—Side view of a set of artificial teeth, showing the manner of putting them into the mouth; *a*, the upper teeth placed in the mouth with the spring, *c*, projecting forward; *b*, the lower teeth, with the forefinger, *d*, placed on the masticating surface, and bending the spring slightly backwards; *e*, the thumb.**

pressed a little backwards, so as to make a backward curve, extending through the whole length of the spring, and similar in direction, though less in degree, to that which they assume when the teeth are properly placed in the mouth. Having grasped the lower teeth, and got the springs in the proper position, in the manner described and shown in fig. 10, they may, without difficulty, be pressed into the mouth.

In some sets it will be found more convenient to place the lower teeth in the mouth first. In such a case the upper ones should be held in the left hand, while with the right the lower teeth are laid upon the gums. Having done this, the thumbs should be placed on the masticating surface of the upper teeth,

Fig. 11.

Fig. 11.—Side view of set of teeth, showing the manner of putting them into the mouth when the lower teeth are first placed: *b*, the lower teeth already placed in the mouth; *c*, the spring projecting forwards and upwards; *a*, the upper teeth; *d*, the forefinger; *e*, the thumb placed on the masticating surface, so that in pressing the upper teeth into the mouth, the spring will assume its proper position.

in which act the springs at their middle part should be pressed backwards towards the mouth, in the same manner as I have described when speaking of the lower teeth, when they are the last to be introduced. The teeth being grasped in the manner shown in fig. 11, and the springs bent backwards in a single curve, they will readily pass into the mouth.

It will be obvious, on again referring to figs. 10 and 11, that whether the upper or lower teeth be placed first, the ends of the springs attached to that half of the set which is first put in the mouth will come forward in the opposite direction to that which they hold when both parts are in the mouth. But it not very unfrequently happens that the springs will not move forward in the manner described with equal readiness in two halves of the set. In the one half they may come forward without difficulty, while in the other they are subject to considerable lateral flexture if the attempt be made, endangering the integrity of the springs. Hence this point should be ascertained, and that division of the set should always be introduced first, on which the springs can readily come forward. If the other division be first placed, the springs will be bent laterally, and, on introducing the other part of the teeth, will not go back into their proper position; the curve backwards will commence at the termination of the pin of the loop, in the manner shown at *d* in fig. 9, and the springs will either be broken off at that point or will be permanently bent.

In removing the teeth from the mouth that division on which the springs will not move forward should be first taken out, otherwise they will be injured almost as badly as though the same part had been first introduced.

A spring which has once been permanently bent can never be restored to its former condition, and from that time will act but imperfectly.

I may here remark that springs, however carefully manufactured and used, will sometimes break suddenly and without any obvious cause; hence those who are dependent on them should have a second pair, unless they have a second set of teeth.

It will readily be seen from what has been already said, that it is of great consequence the spring should move freely on the swivels, otherwise it will be almost impossible to avoid an accident.

It is of great importance that you should know how to preserve false teeth, for in the absence of proper attention they are soon destroyed, and still sooner become offensive. The wearer often seems singularly unconscious of the offensive odor which arises from neglected teeth—not so, however, the bystander; he is almost poisoned by the offensive breath of his neighbor. Dentine is used to some extent in the construction of most sets of teeth, and this substance, you are aware, if neglected, is soon acted on by the saliva, and gradually suffers decomposition: hence arises the bad breath.

I have told you on several occasions, and I again repeat, that dentine, when highly polished, resists the solvent action of the saliva, and, therefore, is not subject to decomposition. The wearer should pay great attention to this point. The surfaces of the teeth (whatever be their kind) should be well brushed with a little precipitated chalk, once or twice a day; and, after brushing, rubbed with a dry soft towel, or handkerchief, or a piece of wash-leather. By these means a beautifully polished surface may be maintained. When not in the mouth, teeth which have dentine, or natural teeth entering into their construction, should be kept in a well-stoppered glass jar, filled with two-thirds of spirits of wine, and one-third of water. Such a jar is shown in fig. 12. The antiseptic quality of the spirit aids much in preserving the dentine, and moreover keeps the teeth sweet. By great attention, cleaning, and emersion in spirits of wine, when out of use, artificial teeth will last quite as long again as they would if these means were neglected. If, however, gold and mineral teeth are alone used, then it will be sufficient to place them in water, after they have been well cleaned. Artificial teeth cannot be too well kept, but they can be very easily, and frequently are, too ill kept. If the base be gold, and the teeth mineral, still they should be well cleaned each day: if the base be gold, and the teeth dentine, there is yet greater need of frequent and careful cleaning. If the base be of den-

Fig. 12.

tine, and the teeth natural teeth, the piece will soon be destroyed if cleaning be neglected. Nothing short of never removing artificial teeth from the mouth should be more strongly deprecated than the habit some people have of taking them out only one or twice a week, and at other times cleaning them in the mouth. They cannot be well cleaned when in the mouth, and the surface of the mouth cannot remain healthy when perpetually covered. For it must be borne in mind that the gums, like skin, are covered with a membrane composed of minute adherent scales called epithelium, and that it is the nature of this tissue to be perpetually forming below, while it is suffering perpetual loss from its surface. The external scales of which it is composed are rubbed off by the friction of the tongue and the food. Now, if the epithelium be perpetually covered by the base of artificial teeth, the formation will still go on, but the loss from the surface is retarded. The outer epithelial scales may separate, but cannot escape from the surface; they, therefore, accumulate under the base, and there become highly offensive. After a while, the mucous membrane inflames, and the

**Fig. 12.—A glass jar, with parallel sides, and stopper accurately ground, suitable for containing artificial teeth.**

development of epithelium is suspended or vitiated; the scales no longer adhere to each other to form a membrane. If the teeth be removed after the mouth has got into this condition, the surface which has been covered will be found red and vascular, and will bleed on the slightest touch. The fitting surface of the teeth will be coated with a highly offensive white cheese-like matter.

Artificial teeth should not, as a habit, be worn during the night, unless their presence is necessary to the comfort of the patient, or for the preservation of the remaining natural teeth. In either case it is desirable that the patient should have a set for the night—a set with a much smaller base than those used for mastication; and, when practicable, a piece fitted to one jaw only, and extended to the opposite jaw for the gums to close on. All that is required of night-teeth is, that they shall keep the jaws apart. The surface of the gums is, in the natural state of the mouth, uncovered, bathed with saliva, and subject to friction; it is desirable, therefore, that it should be left free eight hours out of the twenty-four; and, if some part must be covered even during night-time, let that be as small in extent as possible.

It will be inquired, at what time of life, and under what circumstances, recourse should be had to artificial teeth, how much may reasonably be expected of them, and how long they will last? Artificial teeth should be adopted whenever the want of teeth is felt, whenever articulation becomes imperfect, or when mastication can no longer be performed by molar teeth. I say molar teeth, because some persons, when the grinders are lost, masticate with the front teeth, in which case the incisors are soon worn down, or the upper ones are driven outwards and loosened by the action of the lower front teeth upon their posterior surface; and thus they, by being forced into use for a purpose for which they are not fitted, become speedily destroyed.

If the wearer be a person of average perseverance, and average conformation of mouth, he may expect to have articulation perfectly restored, and mastication of ordinary food rendered effective, by using well-designed and well-made artificial teeth.

Then, as regards the durability of artificial teeth. This will vary with individuals, the variation depending on the state of the saliva, the care with which they are cleaned and kept and used, and upon the material used in their construction; also in a great degree on the manner in which they are made, whether ill or well. A well-made set will last out two ill-made sets. One or two teeth on a gold base will last an indefinite time—ten, fifteen, or twenty years, or they may require renewal in two years, their durability depending on the state of the adjoining natural teeth.

Some people will wear a complete set ten years without renewal, while others wear them down in eighteen months. From three to four years is a fair average wear.

The last subject for consideration is the effects of artificial teeth on the natural teeth which remain in the mouth at the time of their adoption.

It is sometimes stated that artificial destroy the natural teeth to which they are attached by bands; but, I think, seldom with truth, unless the adopted teeth are badly constructed, or the wearer is careless about keeping the teeth and mouth in a clean and healthy condition. That teeth encircled by bands occasionally decay is quite true, but they might have decayed had they not been so encircled; and this is the more probable, as the adjoining teeth have already been lost by decay, in nineteen cases out of twenty were artificial teeth are required. Indeed, in estimating the effects of artificial teeth, it must be constantly borne in mind that the natural teeth have shown a predisposition to disease. Of course, this observation does not apply in those cases in which the teeth have been lost by accidental violence; and it is found that, in those instances, the teeth encircled by bands very seldom suffer.

Supposing, however, it were an established fact that teeth around which bands pass for retaining artificial teeth are thereby seriously damaged; yet, in many cases, it would not be inexpedient to employ them. Frequently, the encircled tooth has no other value than that of supporting artificial teeth, on account of the opposing natural tooth of the opposite jaw being absent: hence it is ineffective as an organ of mastication, and valueless unless used to support or oppose artificial teeth.

But to repeat what I have before stated, mastication is necessary for the maintenance of health. If, therefore, so many of the natural teeth have been lost that it can no longer be efficiently performed, artificial teeth should be adopted, even at the cost of the remaining natural teeth. For when these are lost the artificial ones may be extended, and the patient is not in a worse position than when they remained. But, as I before observed, the use of a partial set of artificial does not necessarily involve the premature loss of the remaining natural teeth. On the contrary, their durability is in many cases very considerably increased.

Having given a general outline of the construction of artificial teeth, together with a more detailed account of those points connected with their management with which it is important that those who are interested, either directly or indirectly, in their use should be made acquainted, I shall conclude the lecture.

## Figures and Tables

**Fig. 1. f1:**
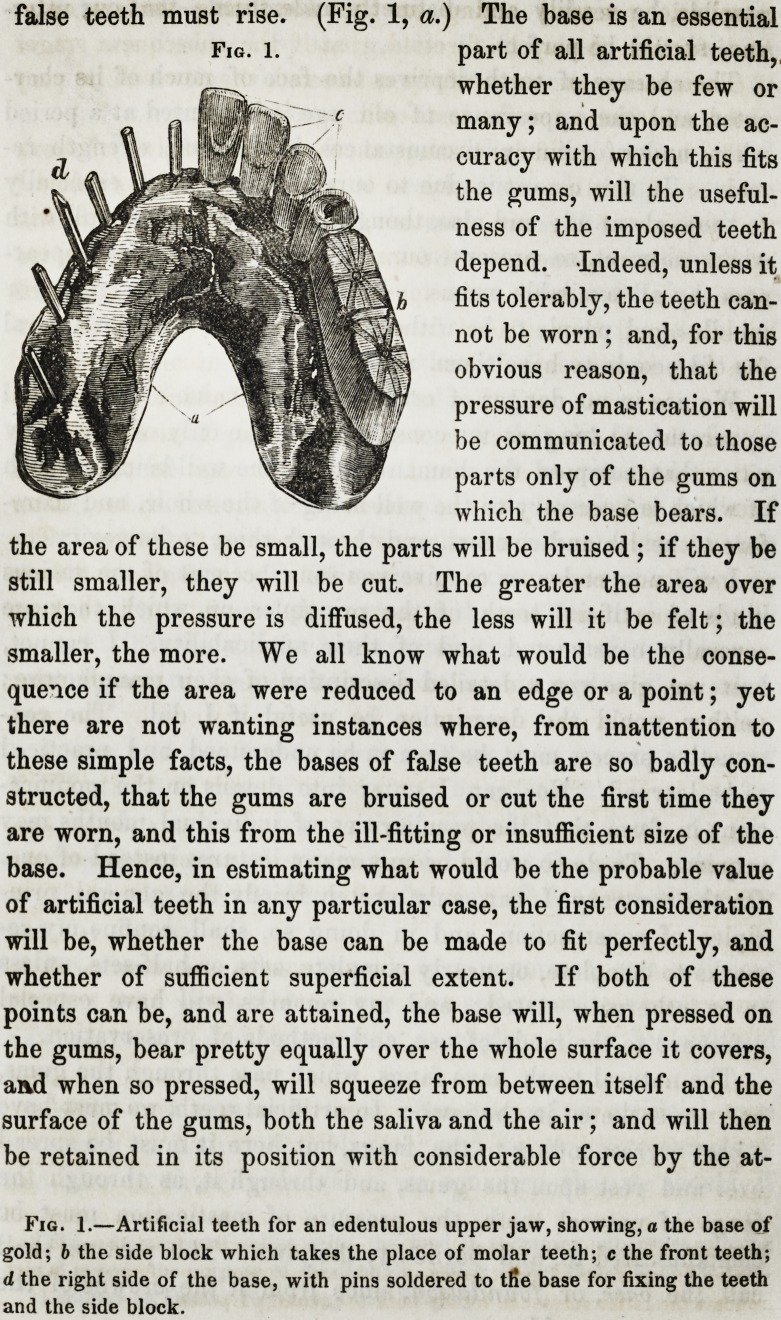


**Figure f2:**
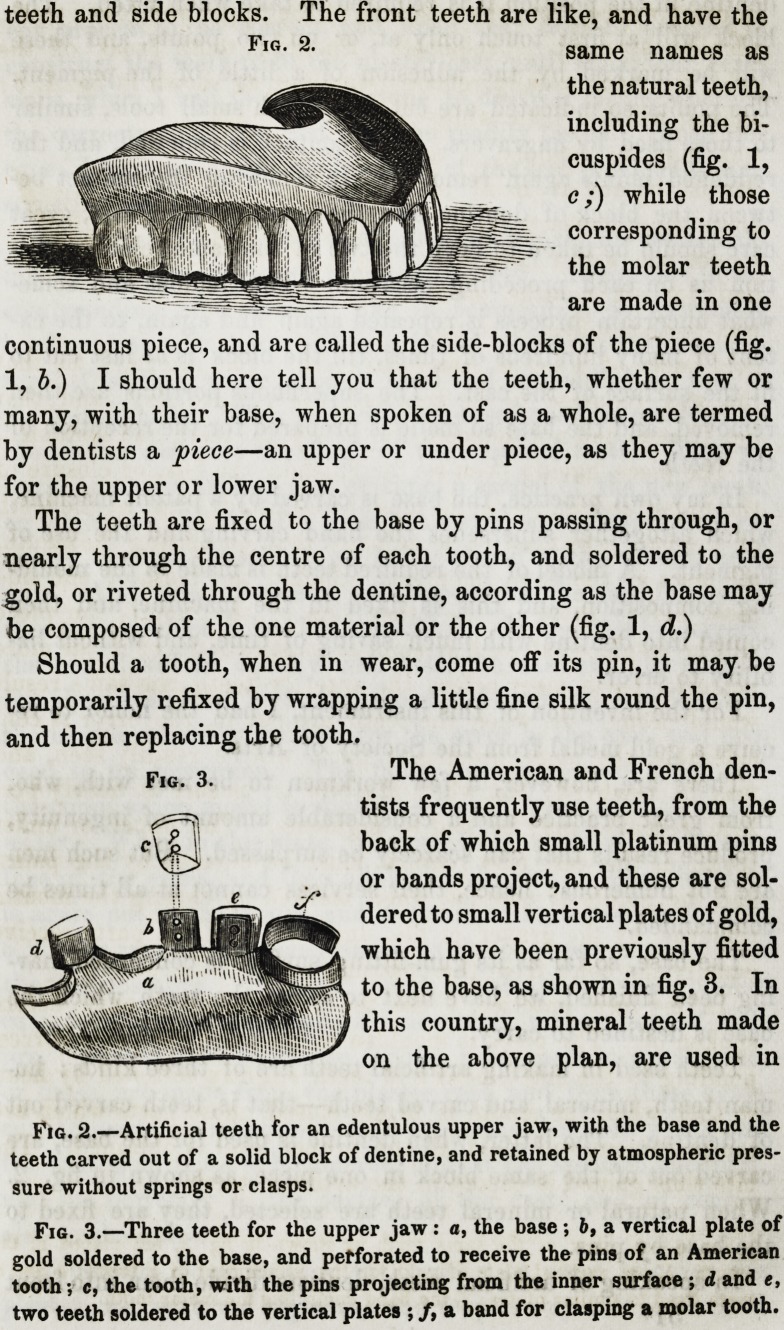


**Fig. 4. f3:**
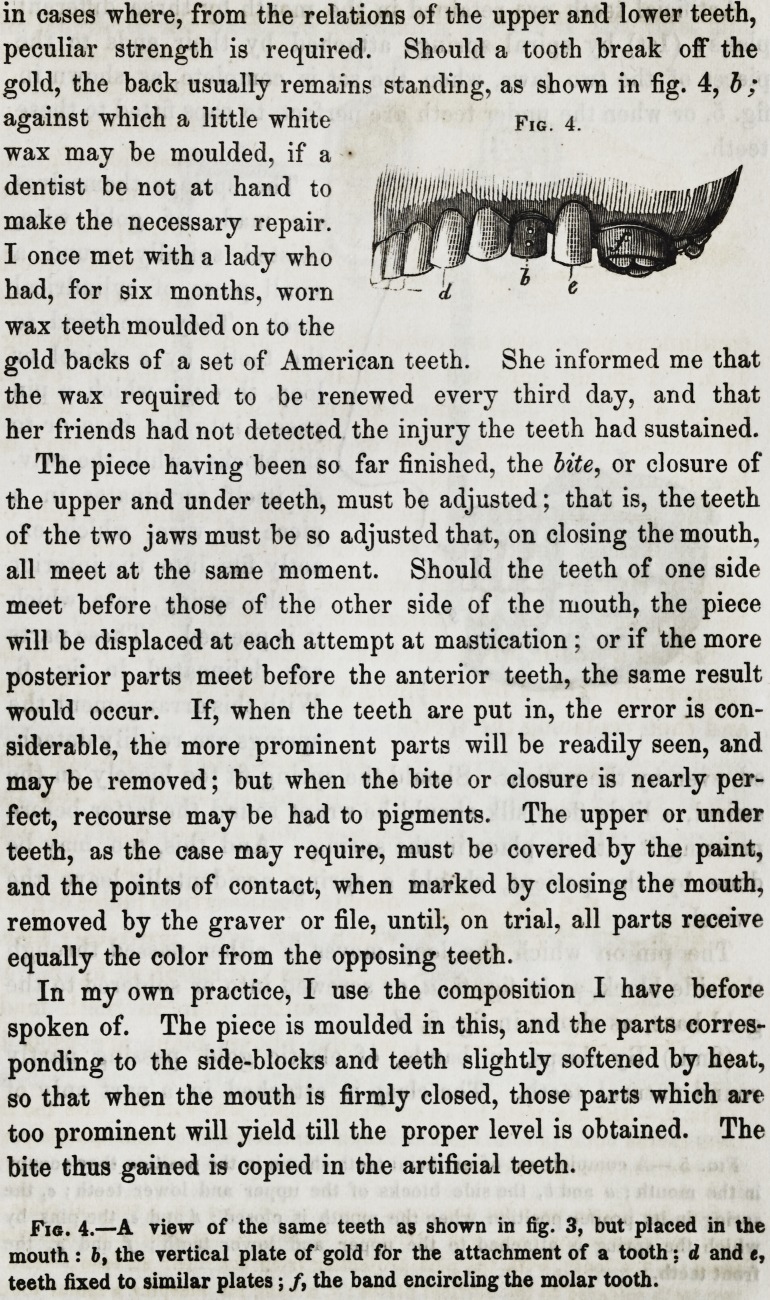


**Fig. 5. f4:**
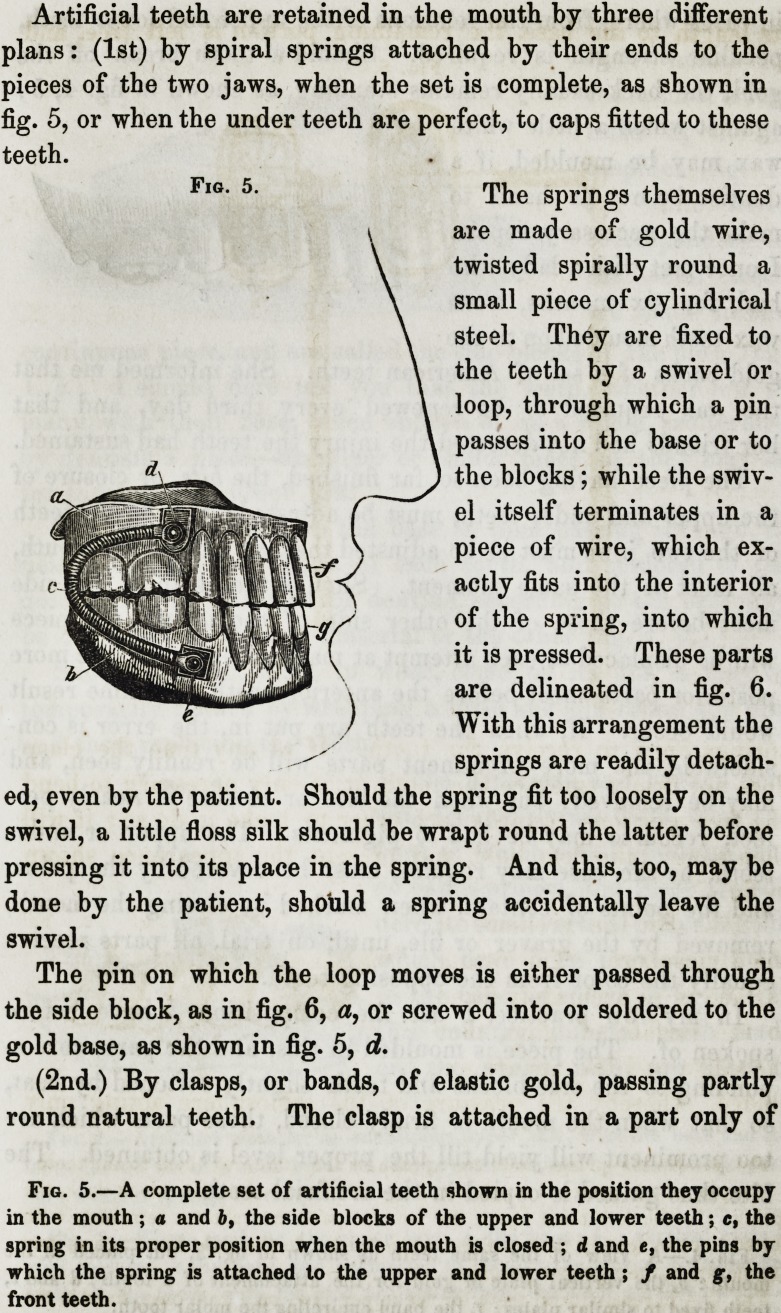


**Figure f5:**
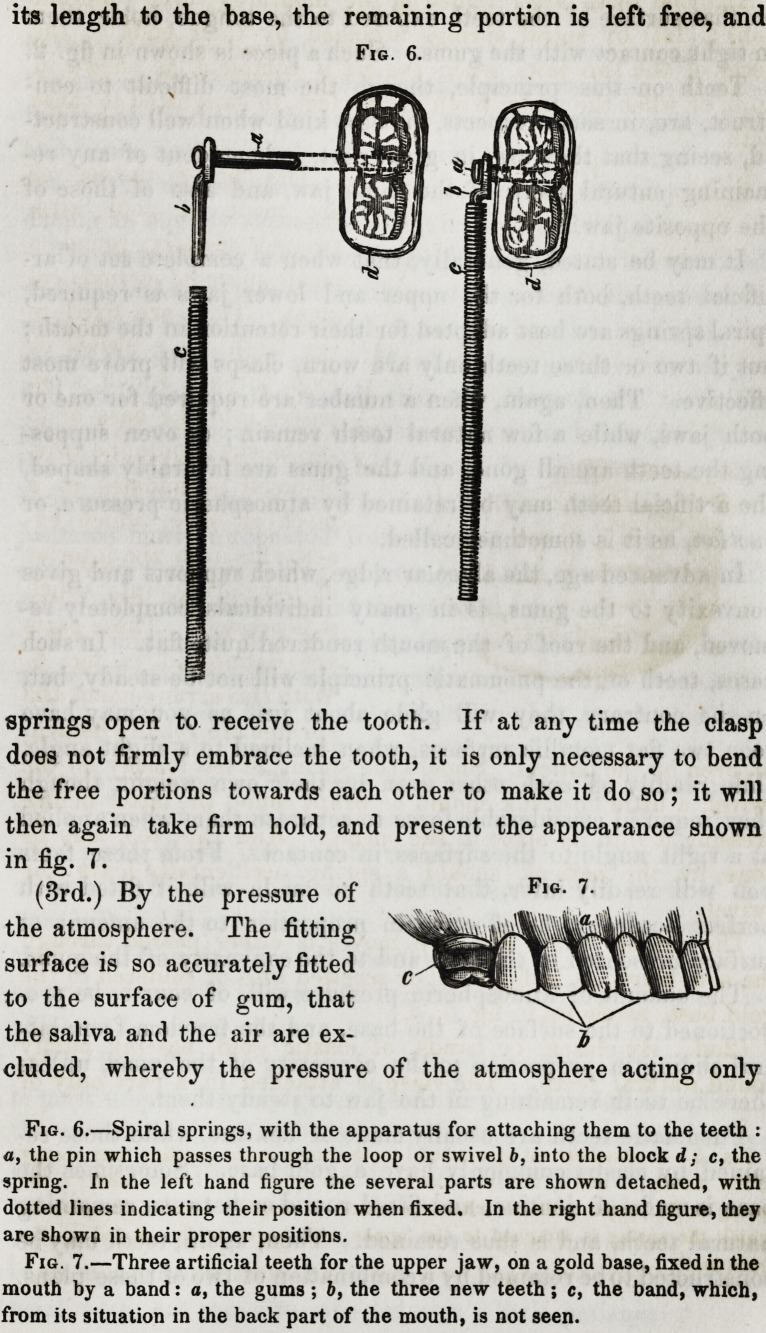


**Fig. 8. f6:**
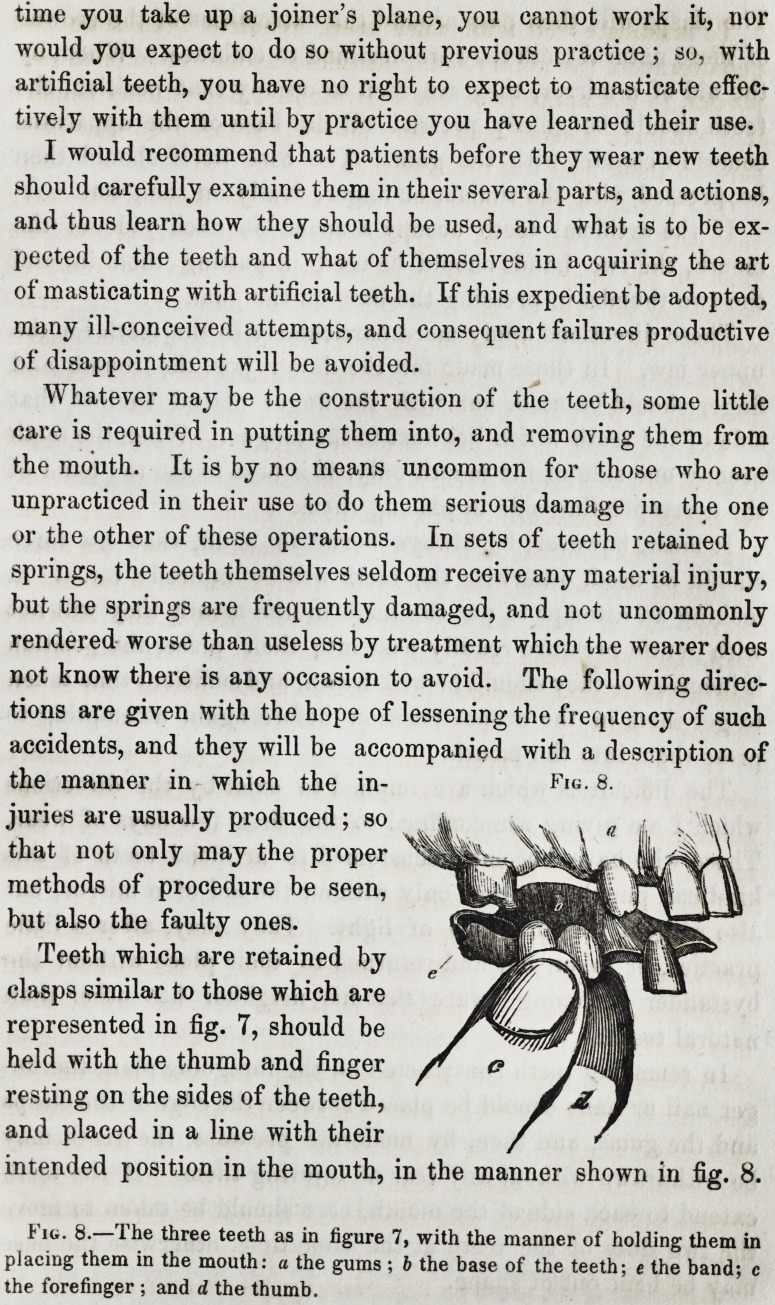


**Fig. 9. f7:**
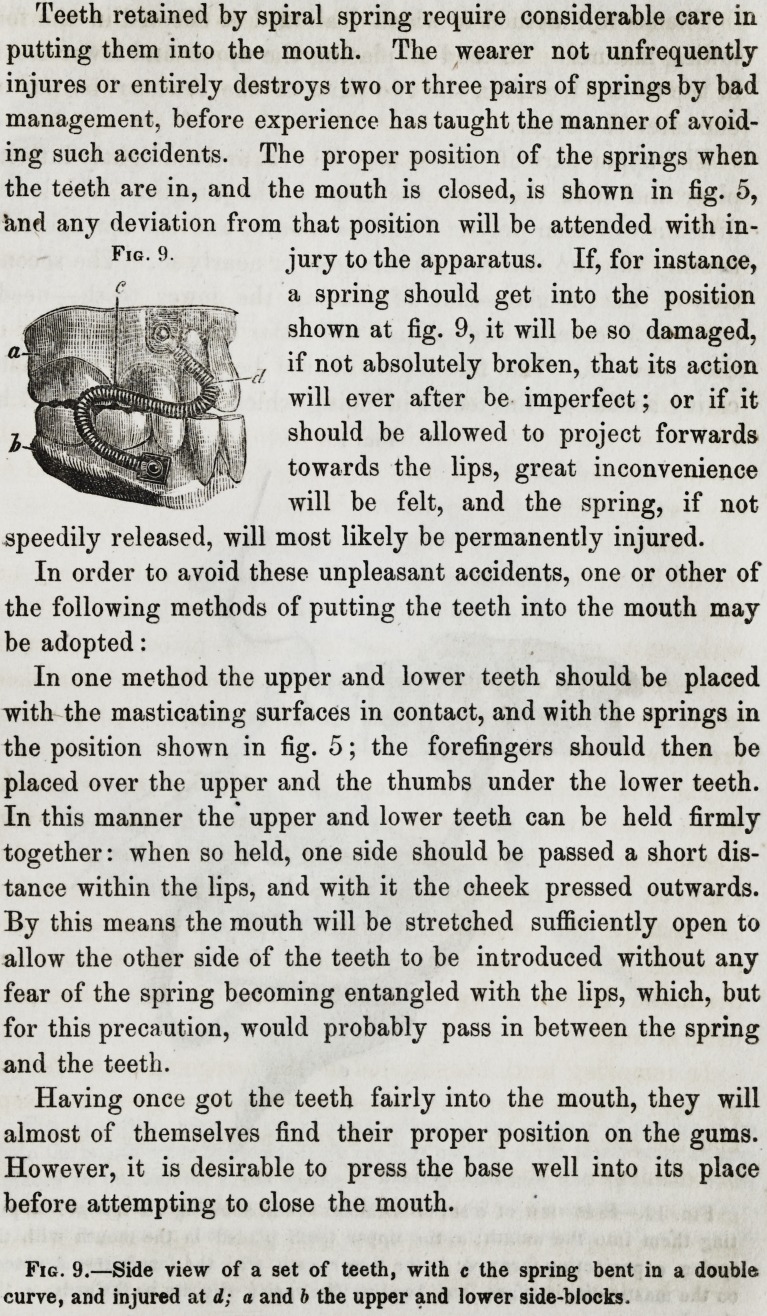


**Fig. 10. f8:**
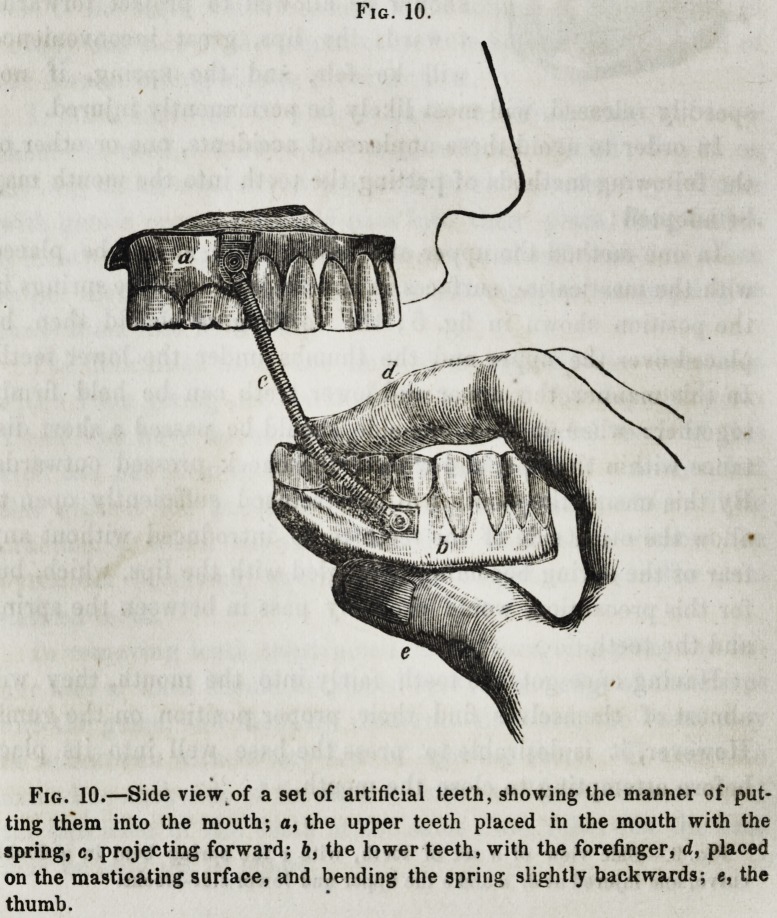


**Fig. 11. f9:**
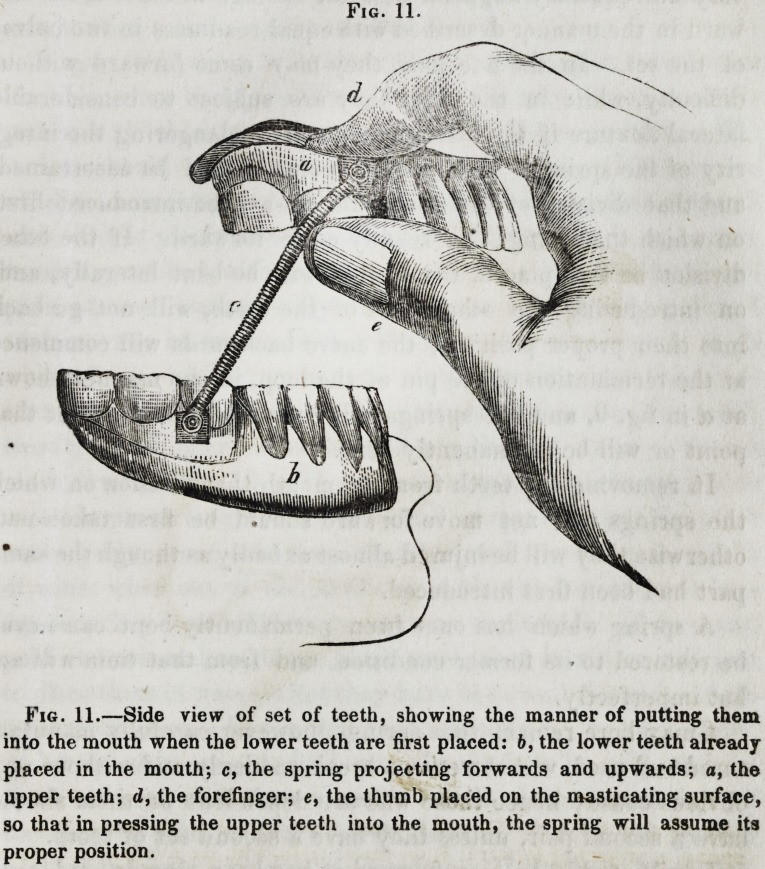


**Fig. 12. f10:**